# Supplementary material for: Axon-like protrusions promote small cell lung cancer migration and metastasis
Source: eLife. 2019 Dec 13;8:e50616. doi: 10.7554/eLife.50616 (PMC6940020; doi:10.7554/eLife.50616)
Supplement: Supplementary file 1. [file elife-50616-supp1.docx]

| **Key Resources Table** | | | | |
| --- | --- | --- | --- | --- |
| **Reagent type (species) or resource** | **Designation** | **Source or reference** | **Identifiers** | **Additional information** |
| gene (M*us musculus*) | *Gap43* | www.ensembl.org | [ENSMUSG00000047261](http://www.ensembl.org/id/ENSMUSG00000047261) |  |
| gene (M*us musculus*) | *Fez1* | www.ensembl.org | [ENSMUSG00000032118](http://www.ensembl.org/id/ENSMUSG00000032118) |  |
| gene (M*us musculus*) | *Cxcr4* | www.ensembl.org | [ENSMUSG00000045382](http://www.ensembl.org/id/ENSMUSG00000045382) |  |
| gene (M*us musculus*) | *Nfib* | www.ensembl.org | [ENSMUSG00000008575](http://www.ensembl.org/id/ENSMUSG00000008575) |  |
| gene (M*us musculus*) | *Mapt* | www.ensembl.org | [ENSMUSG00000018411](http://www.ensembl.org/id/ENSMUSG00000018411) | Also known as Tau |
| gene (M*us musculus*) | *Tubb3* | www.ensembl.org | [ENSMUSG00000062380](http://www.ensembl.org/id/ENSMUSG00000062380) | Also known as Tuj1 |
| gene (M*us musculus*) | *Dclk1* | www.ensembl.org | [ENSMUSG00000027797](http://www.ensembl.org/id/ENSMUSG00000027797) |  |
| genetic reagent (M*us musculus*) | *Trp53^flox^, Rb1^flox^, p130^flox^,* and *R26^mTmG^* | Muzumdar et al., 2007; Schaffer et al., 2010; Denny et al., 2016 | RRID:MMRRC_043692-UCD |  |
| genetic reagent (M*us musculus*) | NOD.Cg-PrkdcscidIl2rgtm1Wjl/SzJ (NSG) | The Jackson Laboratories | RRID:IMSR_JAX:005557 |  |
| cell line (*Homo-sapiens*) | 293T | ATCC | CRL-11268 |  |
| cell line (*Homo-sapiens*) | NCI-H446 | ATCC | HTB-171 |  |
| cell line (*Homo-sapiens*) | NCI-H1694 | ATCC | CRL-5888 |  |
| cell line (*Homo-sapiens*) | NCI-H2081 | ATCC | CRL-5920 |  |
| cell line (*Homo-sapiens*) | NCI-H69 | ATCC | HTB-119 |  |
| cell line (*Homo-sapiens*) | NCI-H82 | ATCC | HTB-175 |  |
| biological sample (*Homo-sapiens*) | Patient Derived Xenograft (PDX) LX102 | Leong et al.,  2014 |  |  |
| biological sample (*Homo-sapiens*) | Patient Derived Xenograft (PDX) LU86 | Saunders  et al., 2015 |  | A gift from Stemcentrx |
| biological sample (*Homo-sapiens*) | Patient Derived Xenograft (PDX) LU102 | Saunders  et al., 2015 |  | A gift from Stemcentrx |
| cell line (M*us musculus)* | KP22 | Denny et al., Cell. 2016 |  | Mouse primary cell line derived from *Rb^f/f^; Trp53^f/f^* |
| cell line (M*us musculus)* | 16T | Denny et al., Cell. 2016 |  | Mouse primary cell line derived from *Rb^f/f^; Trp53^f/f^* |
| cell line (M*us musculus)* | N2N1G | This paper. See Methods. This cell line was derived from a mouse SCLC tumor. |  | Mouse primary cell line derived from *Rb^f/f^; Trp53^f/f^; p130^f/f^* |
| cell line (M*us musculus)* | 6PF | This paper. See Methods. This cell line was derived from a mouse SCLC tumor. |  | Mouse primary cell line derived from *Rb^f/f^; Trp53^f/f^; p130^f/f^* |
| transfected construct (M*us musculus*) | Gap43 shRNA#1 | Sigma-Aldrich | CTGTAGACGAAG  CCAAACCTA | Lentiviral  construct to  transfect and express  the shRNA. |
| transfected construct (M*us musculus*) | Gap43 shRNA#2 | Sigma-Aldrich | GAGTCCACTTTCC  TCTCTATT | Lentiviral  construct to  transfect and express  the shRNA. |
| transfected construct (M*us musculus*) | Fez1 shRNA#1 | Sigma-Aldrich | GAGGACCTCGTGA  ATGAATTT | Lentiviral  construct to  transfect and express  the shRNA. |
| transfected construct (M*us musculus*) | Fez1 shRNA#2 | Sigma-Aldrich | CGTGAATGAATTT  GATGAGAA | Lentiviral  construct to  transfect and express  the shRNA. |
| transfected construct (M*us musculus*) | Cxcr4 shRNA#1 | Sigma-Aldrich | ACTTCTGATAACTA  CTCTGAA | Lentiviral  construct to  transfect and express  the shRNA. |
| transfected construct (M*us musculus*) | Cxcr4 shRNA#2 | Sigma-Aldrich | GTGTTTCAATTCC  AGCATATA | Lentiviral  construct to  transfect and express  the shRNA. |
| transfected construct (M*us musculus*) | GFP  shRNA | Sigma-Aldrich | GCAAGCTGACCCTG  AAGTTCAT | Lentiviral  construct to  transfect and express  the shRNA. |
| transfected construct (M*us musculus*) | Dclk1  shRNA#1 | Sigma-Aldrich | GCCCTTTAAGAAG  CTGGAGTA | Lentiviral  construct to  transfect and express  the shRNA. |
| transfected construct (M*us musculus*) | Dclk1  shRNA#2 | Sigma-Aldrich | GACCGCTACTTCA  AAGGAATT | Lentiviral  construct to  transfect and express  the shRNA. |
| transfected construct (M*us musculus*) | Dcc  shRNA#1 | Sigma-Aldrich | GCCAGCATAAGAACA  GGAAAT | Lentiviral  construct to  transfect and express  the shRNA. |
| transfected construct (M*us musculus*) | Dcc  shRNA#2 | Sigma-Aldrich | CCCAAGGATCTGACAG  TCATT | Lentiviral  construct to  transfect and express  the shRNA. |
| transfected construct (M*us musculus*) | Tiam1  shRNA#1 | Sigma-Aldrich | CGGAATTTGGTGTCGG  ATATT | Lentiviral  construct to  transfect and express  the shRNA. |
| transfected construct (M*us musculus*) | Tiam1  shRNA#2 | Sigma-Aldrich | GCCGCTGATAATTACG  GGTTT | Lentiviral  construct to  transfect and express  the shRNA. |
| transfected construct (M*us musculus*) | Dbn1  shRNA#1 | Sigma-Aldrich | GCAGTCTATCTTTGGTG  ACCA | Lentiviral  construct to  transfect and express  the shRNA. |
| transfected construct (M*us musculus*) | Dbn1  shRNA#2 | Sigma-Aldrich | CCCAGACCAGATTGTA  GCTTA | Lentiviral  construct to  transfect and express  the shRNA. |
| transfected construct (M*us musculus*) | Dcx  shRNA#1 | Sigma-Aldrich | CGGCTGTAATTGGTGGT  TGTA | Lentiviral  construct to  transfect and express  the shRNA. |
| transfected construct (M*us musculus*) | Dcx  shRNA#2 | Sigma-Aldrich | CGCGTGCTTCTCAACAA  GAAA | Lentiviral  construct to  transfect and express  the shRNA. |
| transfected construct (M*us musculus*) | Fscn1  shRNA#1 | Sigma-Aldrich | CTACAATAAGGTGGCT  CTCAA | Lentiviral  construct to  transfect and express  the shRNA. |
| transfected construct (M*us musculus*) | Fscn1  shRNA#2 | Sigma-Aldrich | CCGTTCCAGTTACGATG  TCTT | Lentiviral  construct to  transfect and express  the shRNA. |
| transfected construct (M*us musculus*) | Lrp8  shRNA#1 | Sigma-Aldrich | CGCAGCAATCAGCAACT  ATGA | Lentiviral  construct to  transfect and express  the shRNA. |
| transfected construct (M*us musculus*) | Lrp8  shRNA#2 | Sigma-Aldrich | CCCATCTCTGATCTTCA  CGAA | Lentiviral  construct to  transfect and express  the shRNA. |
| transfected construct (M*us musculus*) | Map1b  shRNA#1 | Sigma-Aldrich | CCGAGTTAGACATCAA  AGAT | Lentiviral  construct to  transfect and express  the shRNA. |
| transfected construct (M*us musculus*) | Map1b  shRNA#2 | Sigma-Aldrich | CGGATTCAACATGCTCA  TCAA | Lentiviral  construct to  transfect and express  the shRNA. |
| transfected construct (M*us musculus*) | Reln  shRNA#1 | Sigma-Aldrich | CCGTGGAAATTGTGATC  TGTT | Lentiviral  construct to  transfect and express  the shRNA. |
| transfected construct (M*us musculus*) | Reln  shRNA#2 | Sigma-Aldrich | CCAGGATACATGATGCA  ATTT | Lentiviral  construct to  transfect and express  the shRNA. |
| transfected construct (M*us musculus*) | Vldlr  shRNA#1 | Sigma-Aldrich | CCGAGTCTGATCTTCAC  TAAT | Lentiviral  construct to  transfect and express  the shRNA. |
| transfected construct (M*us musculus*) | Vldlr  shRNA#2 | Sigma-Aldrich | CGACGAGAAGAACTGTG  TAAA | Lentiviral  construct to  transfect and express  the shRNA. |
| antibody | Anti-mouse-Tuj1  (Mouse monoclonal) | Biolegend | 801213  RRID:AB_2728521 | IHC/IF (1:500) |
| antibody | Anti-mouse-Tau  (Rabbit polyclonal) | Dako | A0024  RRID:AB_10013724 | IHC/IF (1:1000) |
| antibody | Anti-mouse-Map2  (Rabbit polyclonal) | EMD Millipore | AB5622  RRID:AB_91939 | IHC/IF (1:500) |
| antibody | Anti-GFP  (Chicken polyclonal) | Abcam | ab13970  RRID:AB_300798 | IHC/IF (1:500) |
| antibody | Anti-human/mouse-GAP43  (Rabbit polyclonal) | Abcam | ab16053  RRID:AB_443303 | IHC (1:500) |
| antibody | Anti-human/mouse-CGRP  (Mouse monoclonal) | Sigma-Aldrich | C7113  RRID:AB_259000 | IHC/IF (1:200) |
| antibody | Anti-human/mouse-UCHL1  (Rabbit polyclonal) | Sigma-Aldrich | HPA005993  RRID:AB_1858560 | IHC/IF (1:500) |
| antibody | Anti-human/mouse-Fez1  (Rabbit monoclonal) | Cell Signaling | 42480  RRID:AB_2799222 | WB (1:1000) |
| antibody | Anti-human/mouse-Hsp90  (Mouse monoclonal) | BD Transduction Laboratories | 610418  RRID:AB_397798 | WB (1:1000) |
| antibody | secondary HRP-conjugated anti-mouse  (Goat polyclonal) | Santa Cruz Biotechnology | sc-2005  RRID:AB_631736 | WB (1:2000) |
| antibody | secondary HRP-conjugated anti-rabbit  (Goat polyclonal) | Santa Cruz Biotechnology | sc-2030  RRID:AB_631747 | WB (1:2000) |
| commercial assay or kit | Silicone inserts | ibidi | 80209 |  |
| chemical compound, drug | DiI  red fluorescent tracer | Thermo Fisher Scientific | D282 |  |
| software, algorithm | Graphpad Prism8 | Graphpad | Prism8  RRID:SCR_002798 |  |
| software, algorithm | Metascape | Zhou et al., 2019 | metascape.org  RRID:SCR_016620 |  |
| software, algorithm | Cancer Dependency Map Portal | Tsherniak et al., 2017 | [depmap.org/portal/](https://depmap.org/portal/) |  |
| other | Tissue microarrays  LC818a | US Biomax | LC818a |  |
| other | Tissue microarrays  /sections | This paper. See Methods. These samples were obtained from the Pathology department. | Human SCLC metastases sections from Stanford Hospital |  |
